# Clinical and bacteriological profile of abdominal surgical site infections in an Indian Hospital

**DOI:** 10.6026/97320630018962

**Published:** 2022-10-31

**Authors:** Kumar Ratnesh, Somen Jha, Anamica Arya

**Affiliations:** 1 PG Department of General Surgery, Jawaharlal Nehru Medical College and Hospital, Bhagalpur, Bihar, India

**Keywords:** Abdomen, Surgical Site Infection, bacterial profile

## Abstract

This study was carried out to assess the clinical and bacterial profiles of abdominal surgery site infections in a tertiary care hospital.Samples recovered from infected wounds at abdominal surgery sites were processed using highly advanced microbiological procedures. To process these samples, the most recently accepted standard CLSI guidelines were used. Antimicrobial vulnerability was investigated using a modified Kirby-Bauer disc diffusion method.97 samples were collected from 83 patients who had proven evidence of infections at abdominal surgery sites. It was found that 97.5% of the total samples had evidence of significant growth of bacteria and bacterial isolates obtained were 88 in number. Staphylococcus aureus was the most often isolated bacterium, accounting for 51.52% of total samples. The second most prevalent germ isolated was Escherichia coli, which accounted for 24.13% of total samples. It was concluded that the high prevalence of infections at the surgical sites of abdomen in our study highlights the importance of providing high-quality surgical care that considers the features of the host, environment, and microorganisms before performing any surgery.

## Background:

Nosocomial infections are common, with infections at abdominal surgery sites being one of the most common causes (2-20%). They are more common following abdominal surgery. They are to blame for considerable mortality and morbidity as well as rising treatment costs and hospital stays. Even in institutions with the most up-to-date facilities, infection at the abdominal surgical site remains a serious issue despite recent technical breakthroughs in preventing infection and surgical techniques. [1, 2] Infections at surgical sites are often caused by endogenous and/or exogenous microorganisms that enter the site during surgery (primary infection) or soon afterwards (secondary infection). More severe complications from primary infections may not appear until around 5-8 days following surgery. [3-4] Necrotizing infections are extremely uncommon at abdominal surgery sites, with the great majority of infections being uncomplicated infections merely affecting the skin as well as subcutaneous tissue. A surgical wound that is contaminated manifests as sensitivity, pain, higher body temperature, increased redness, swelling, along with pus production. [5-6] Several patient-specific factors, such as advanced age, poor nutritional status, the presence of a pre-existing infection, and the presence of other co-morbidities, may considerably impact the likelihood of an infection developing at the site of abdominal surgery. [7-8] Surgical site infections may result from careless techniques, extended operating hours, improper sterilization of equipment, and the use of filthy surgical instruments. "Other factors, such the pathogenicity and virulence of the organisms involved, the physiological condition of the tissue proximal to the wound, and the immunological coherence of the host, also play a role in whether or not an infection occurs. At each location of abdominal surgery, bacterial testing has confirmed the presence of illness. The research also shows that the etiological agents for these infections at surgical sites vary depending on the region, kind of operation, surgeon, hospital, and even the specific ward". [9-10] In hospitals, Gram negative microbes have become a more frequent source of severe infections in recent years. The incorrect usage of broad-spectrum antibiotics has exacerbated the problem of antimicrobial drug resistance. In developing nations, inadequate infection control procedures, overcrowded hospitals, in addition to incorrect antibiotic use exacerbate the problem. [11-12] Therefore, it is of interest to explore the clinical as well as microbiological aspects of abdominal surgery site infections at a tertiary hospital.

## Methods and materials:

This hospital based research was carried out for duration of eighteen months. Before collecting the sample, the Organizational Ethical Committee approved the study. The review population consisted of 83 patients with diseases affecting the careful regions of the abdomen, and they came from our clinic's various specialized units (muscular unit, general a medical procedure unit, ophthalmology medical procedure unit, obstetrics and gynecology specialized unit, and otorhino laryngology specialized unit). Selected focus group participants were adults (defined as those older than 14 years of age) with watery or sero purulent discharge from surgical injuries and/or associated indications of sepsis (redness, in duration at the careful injuries, irritation, torment, expanded nearby temperature). Stitch abscess patients, those with cellulitis-contaminated wounds, and those without waste were not included in the analysis. Patients provided a detailed clinical history, recalling information such as age, orientation, sickness type, finding, kind of activity, duration of medical treatment, anti-toxins used, and co-grim conditions. Culture and sensitivity of lesions were acquired from 83 patients who had infections at the abdominal surgery site that were clinically proven. Samples recovered from infected wounds at abdominal surgery sites were processed using highly advanced microbiological procedures. To process these samples, the most recently accepted standard CLSI guidelines were used. Antimicrobial vulnerability was investigated using a modified Kirby-Bauer disc diffusion method. Two pus/wound specimens were aseptically taken from every participant suspected of having infections of surgical wounds at the abdomen using sanitized cotton balls. One swab was used to create gram stained preparations for a preliminary diagnosis. The other sample was planted on Mac Conkey agar culture media and five percent sheep blood agar culture plates, maintained at 37°C for forty eight hours, and then declared sterile. The colony characteristics and battery of common biochemical assays were used to identify growth on culture plates. Cefoxitin (30g) was used as a diagnostic biomarker to detect methicillin resistance, and the PBP2a slide agglutination test was used to corroborate the finding. "Antibiotic susceptibility was evaluated using reference strains of Staphylococcus aureus (according to ATCC 25923), Escherichia coli (according to ATCC 25922), and Pseudomonas aeruginosa (according to ATCC 27853). Everything we needed, from dehydrated media to reagents to antibiotic discs, came from Hi Media Laboratories Private Limited in Mumbai, India.

## Statistical Analysis:

T-tests, binary logistic regression, and the chi-square test were only few of the statistical tools used to examine the data. An issue with convergence was encountered when utilizing a binomial regression analysis to evaluate antibiotic susceptibility and bacterial isolates from abdominal surgical sites.”The overall RRs and 95 confidence intervals (CIs) were determined by using a simplified Poisson regression method (confidence intervals).All statistical analysis was performed in Stata (version 14).

## Results:

Among 440 patients who presented to various surgical units at our institutions, 18.9% (83) were found to have infections at the surgical sites of abdomen, indicating a high prevalence of surgical site infections. It is safe to say that males made up a larger share of the study population than females did. There were 2.70 males for every female. Infection at the abdominal incision site was more common in individuals aged 50 and above compared to those under 50. The length of an operation was shown to be a major risk factor for SSI, and it was also found that the incidence of infection increased with both the number of surgeries and their length. 83 individuals with signs of infection at abdominal surgery sites provided 97 samples. It was found that 97.5% of the total samples had evidence of significant growth of bacteria and bacterial isolates obtained were 88 in number. When there was recording of details regarding distribution of morphotypes according to age then it was observed that most of the mono microbial variants and poly microbial variants were found in age group greater than 50 years while sterile variants was maximum in age group of 30 to 40 years. The findings were found to be significant statistically (Table 1 - see PDF) Staphylococcus aureus was the most often isolated bacterium, accounting for 51.52% of total samples. Methicillin resistance was found in 81.2% of these Staphylococcus aureus specimens, while Methicillin sensitivity was found in 18.8%. When compared to other types of isolated bacteria, Escherichia coli was the second most common, making about 24.13 percent of all samples taken. Furthermore, Pseudomonas aeruginosa (8.6% of illnesses) and Citrobacter species (8.1% of illnesses) were also important factors. Microorganisms belonging to the Citrobacter koseri species accounted for 37.4% of the total, while microorganisms belonging to the Citrobacter freundii species accounted for 62.5%. 3.1% of the bacterial confines were caused by Klebsiella species microorganisms, while 3.2% were caused by Proteus species microorganisms. Most Klebsiella species diseases (77%) were caused by Klebsiella oxytoca, whereas Klebsiella pneumonia was responsible for 23%. Altogether, Acinetobacter-related microorganisms accounted for 4.91 percent of the total. Acinetobacter baumannii (72.3% of Acinetobacter-caused illnesses) and Acinetobacter lowfii (27.7% of Acinetobacter-caused illnesses) were evenly split. Diseases caused by bacteria belonging to the Proteus species accounted for 3.2% of all cases. There were 48 percent more cases of disease caused by Proteus mirabilis than by Proteus vulgaris, and vice versa. The two were intrinsically linked, and the relationship was substantial. (Table 2 - see PDF) Gram-positive bacteria were shown to be most susceptible to linezolid, teicoplanin, and vancomycin, and most resistant to penicillins and cephalosporins. Penicillin resistance was recorded at 99.67%, while resistance to cephalosporins was reported at 99.11%. Resistance to linezolid, teicoplanin, and vancomycin was below 10%. When testing the susceptibility of gram-negative bacteria to antibiotics, researchers found that the bacteria were most susceptible to meropenem (0.01%), amikacin (17.91%), and piperacillin (36.24%), while they were highly resistant to beta lactam/beta lactamase inhibitor antibiotic combinations (96.35%). In a statistical analysis, the results were judged to be significant. (Table 3, 4 - see PDF)

## Discussion:

Even if cautious procedures have advanced and doctors have a better grasp on the etiology of wound contamination after a medical treatment, specialists nonetheless experience significant amounts of stress while treating illnesses at stomach careful locations. Patients undergoing medical procedures in a clinic atmosphere that is perpetually polluted by microbial germs will be exposed to a gusher of microbiota. Medical procedures performed on the stomach provide a small risk of necrotizing illnesses, even though these infections typically only affect the skin and subcutaneous tissue.[13, 14] Infected surgical wounds cause discomfort and may cause fever, increased redness, edoema and pus to occur. Several patient-specific factors, such as advanced age, poor nutritional state, the presence of prior infections, and other co-morbidities, may increase the risk of infection at the site of abdominal surgery. These infections may be caused by a variety of factors, including sloppy surgery, a lengthy procedure, improper sterilization of surgical equipment, and unclean surgical parts.[15, 16] The present scenario is made worse by the fast and unchecked growth of resistance to the several antimicrobials already in use. Most infections at the sites of abdominal surgery are acquired in the hospital, and the prevalence of these illnesses varies widely from one medical center to the next. The incidence of infection at the site of abdominal surgery is predicted to range from 2.5% to 41.9%.[17,18] Infections at abdominal surgery sites were more common in the present research (18.9%) than in the previous one (13.7%) by Satyanarayana et al. The prevalence of SSI has been estimated to be anywhere between 6.1% and 38.7%, according to several studies conducted in India [10, 12-14].Compared to hospitals in other countries; those in India have one of the highest rates of hospital-acquired infections. There might be a number of reasons for the alarmingly high infection rates in Indian hospitals, including but not limited to improper infection prevention measures, inadequate hand cleanliness, and overcrowding. [19] Compared to other studies, this one had a much higher proportion of male participants (2.70 to 1). The majority of the participants in the research were male. The incidence of infection at the abdominal surgery site was higher in patients over the age of 50 (52.9%), but lower in those under the age of 30 (13.5%). Because older patients heal more slowly, have weaker immune systems, have higher levels of catabolic activity, and are more likely to have co-morbid conditions like diabetes, hypertension, and the like, they are at a significantly higher risk of developing infections at the surgical site following abdominal surgery. [20] The length of the procedure was discovered to be a strong risk factor for infection at the surgical sites of abdomen, and it was revealed that as the sequence and timing of the surgery grew, so did the frequency of infection grew. With an estimated prevalence extending from 4.6 percent to 54.4 percent, Staphylococcus aureus microorganisms, a gram-positive bacteria, is a significant human pathogen and the leading cause of infections at surgical site of abdomen globally. S. aureus predominated (51.5 percent) in the current investigation, which was comparable with data from prior studies. S. aureus infestation is most likely linked to an endogenous source because it belongs to the epidermis and nasal flora, as well as exposure from the outer environment, surgical equipment, or healthcare staff' hands. [21] In this study, S. aureus was the only gram-positive bacterial strain that proved to be very dominating. Considering how commonplace S. hospitals, methicillin-resistant S. aureus, and the spread of hospital-acquired infections involving abdominal surgery sites. Importantly, it is of relevance because certain strains of Staphylococcus aureus (MRSA) are resistant to antibiotics.16.7% of the S. aureus isolates in our investigation exhibited methicillin resistance. This result was similar with the research by Aggarwal et al, which found that 10% of the isolates were resistant to methicillin; however, it differs from the study by Kownhar et al., which found a higher prevalence of 21.7 percent. Eagye et al. & Kaye et al. both reported significantly higher incidences of MRSA of 45 percent and 58.2 percent, respectively. [22] Regardless of methicillin resistance, we discovered that all S. aureus strains were susceptible to vancomycin antimicrobials, teicoplanin antimicrobials, and linezolid antimicrobial. This discovery may have important clinical implications for our hospital's decision-making on antibiotic use. 50 .7% of the all the aerobic isolated bacteria were gram-negative isolates. The most prevalent gram-negative bacteria isolated were E. coli (48.6% of all gram negative infections), P. aeruginosa (17.5% of all gram negative bacteria), and Citrobacter species (18.1 percent of all gram negative bacteria). Many other researchers have also reported making comparable observations. P. aeruginosa, which is the third commonest isolated bacterium in this investigation, has been identified in a small number of publications as the most prevalent isolate in infections at the surgical site of abdomen.[16] The patient's typical indigenous microbial faecal flora could be to blame for the existence of enteric organisms in the microbial isolates from the infection sites. A striking example of sub par hospital hygiene is E. coli infection of the wound. Results of antimicrobial susceptibility tests show that the vast proportion of the bacteria species had high levels of resistance. The most efficient antibiotics against gram-positive bacteria were discovered to be vancomycin antimicrobials, teicoplanin antimicrobials, linezolid antimicrobials, and amikacin antimicrobials. The level of resistance was significantly greater in gram-negative bacteria, and it was discovered that the regularly used medications were more resistant, with an estimated resistance spectrum of 50 percent to 100 percent. Ampicillin antimicrobials, amoxicillin clavulanate antimicrobials, and cefotaxime antimicrobials were among the most refractory medications, whereas meropenem antimicrobials, piperacillin-tazobactam antimicrobials, and amikacin antimicrobials were determined as being the most active antimicrobial medicines. In contrast to earlier investigations, the P.aeruginosa strains identified for this study were shown to be extremely resistant. A major issue on a worldwide scale is the emergence and spread of antimicrobial resistance bacterial species. Recent decades have seen the rise of types of bacteria that are resistant to many classes of antibiotics, and this is often attributed to the widespread use of antimicrobial drugs, which creates a strong selection pressure. [20] Prior to surgery, preventive antimicrobials were given to all participants in our study. The most clinical practice guidelines for prophylactic antibiotics to avoid surgical site infections at abdomen, state that an antimicrobial medication should be given within 60 minutes of surgery and should be stopped shortly after the surgical procedures. But still more than half of our participants underwent preoperative antibiotics therapy more than 6 hours prior to surgery, and nearly all of them received antibiotics afterward. In an effort to stop contamination while they're in the hospitals, most of them were even medicated up to the day of release. Third generation cephalosporins and amino glycosides were the most often utilised combination. The bacterial isolates strains were generally resistant to these drugs, according to the findings of the antimicrobial susceptibility tests. Nearly all of the isolates consistently showed the highest level of ampicillin resistance, and all species with the exception of Proteus species showed this to be statistically meaningful. High resistance may have been a result of our hospital's extensive empirical prescribing of these antibiotics for both therapy and prevention. It's urgent that the antibiotic policy and prescribing recommendations be revised in light of this circumstance. [22] The bacterial isolates we collected from our patients revealed a high degree of resistance to conventional antimicrobials, yet the SSI incidence we saw was comparable to that documented in previous similar studies undertaken in underdeveloped countries like India. But more extensive research is needed to increase their statistical power. Despite the use of prophylactic antimicrobials and cutting-edge surgical and sterilizing procedures, infections at the surgical sites of the abdomen continue to be a major clinical concern. In perspective of pharmaco therapeutic deficits as well as pharmaco economic deficits; they take a heavy toll on the disease of patients and also o healthcare providers. [15, 16] While it may be impossible to eradicate all infections, even those confined to the abdomen, even a significant reduction in the illness rate would have far-reaching positive effects by reducing silent grimness and death and medical services resource waste. This may be aided by providing the highest quality of preoperative, intra operative and postoperative care to the patient. Furthermore, there is compelling evidence that focusing on numerous patient-related risk factors and method-related risk variables may greatly lower the risk of illnesses in the cautious localities of the middle area. In addition, we would implement effective disease control systems and a robust anti-toxin approach, all while prioritizing discretion, safety, and treatment outcomes.[22] One limitation of our study was that we were unable to perform anaerobic bacterial profiling or fungal culture on the skin swabs we collected from infections at abdominal surgical sites. More future research needs to be done in this area.

## Conclusions:

The high prevalence of infections at the surgical sites of abdomen in our study highlights the importance of providing high-quality surgical care that considers the features of the host, environment, and microorganisms before performing any surgery. The cautious use of antibiotics and hospital's adoption of an antibiotic policy is necessary due to the rise in antibiotic resistance.
